# Four pillars of heart failure: contemporary pharmacological therapy for heart failure with reduced ejection fraction

**DOI:** 10.1136/openhrt-2021-001585

**Published:** 2021-03-02

**Authors:** Sam Straw, Melanie McGinlay, Klaus K Witte

**Affiliations:** 1 Leeds Institute of Cardiovascular and Metabolic Medicine, University of Leeds, Leeds, UK; 2 Cardiorespiratory Clinical Services Unit, Leeds Teaching Hospitals NHS Trust, Leeds, UK

**Keywords:** heart failure, systolic, pharmacology, clinical

## Introduction

The past two decades have heralded dramatic improvements in outcomes for people living with heart failure with reduced ejection fraction (HFrEF).^1^ The more widespread implementation of disease modifying pharmacological therapies,^2^ supported by landmark trials of renin-angiotensin system inhibitors^3^ and beta-blockers^4^ have improved longevity despite a background of an ageing and increasingly multimorbid population. Although the benefits of comprehensive pharmacological therapies are clear, the real-world attainment of target doses^5 6^ and utilisation of novel agents such as angiotensin receptor-neprilysin inhibitors (ARNI)^7^ remain low. Furthermore, HFrEF remains a disease associated with significant morbidity and reduced survival relative to those without HFrEF, even after taking into account comorbidities.^8^ Recently, trials have demonstrated improved outcomes in people with HFrEF receiving sodium-glucose co-transporter 2 inhibitors (SGLT2i).^9 10^ However, it is currently unclear how these agents will be used alongside established therapies. Now is therefore an opportune moment to pause and reflect on our current practice, barriers to further progress and how future guidelines might work better for our patients. In this viewpoint we summarise how our current linear approach, on a background of increasingly complex pharmacotherapy has the potential to cause confusion and consequent delays which could lead to even worse attainment of optimal therapies. On the other hand, a more parallel approach to the initiation and optimisation of the Four Pillars of Heart Failure would simplify our approach, yielding benefits for our patients and healthcare systems.

## How did we get here?

Heart failure guidelines are based around inhibition of the renin-angiotensin and sympathetic nervous systems, two fundamental pathways which drive the pathophysiology of HFrEF using ACE inhibitors (ACEi) and beta-blockers. In both European^2^ and American guidelines^11^ additional therapies are recommended for patients who ‘remain symptomatic’ with persistently impaired left ventricular (LV) function despite maximally tolerated doses of ACEi and beta-blockers. These guidelines differ subtly regarding the timing of mineralocorticoid receptor antagonists (MRA) relative to other therapies but are otherwise broadly similar, advocating a linear approach. This attempts to avoid ‘unnecessary’ treatments in patients who ‘respond’ but has several important limitations. First, while guidelines do not stipulate a time interval between alterations to therapy, the need for further assessment and re-evaluation of LV function inevitably results in delays initiating additional agents as well as contributing further follow-up and imaging costs. In clinical practice it typically takes many months before patients receive optimised doses of these medications, and many never do, even where integrated hospital and community care is available.^5^ Second, the barrier of ‘response’ is confusing and misplaced: does ‘response’ mean asymptomatic or merely improved? In our experience, while patients often feel better, they rarely become asymptomatic (NYHA (New York Heart Association) class 1),^12^ an observation supported by real-world data even in those receiving ARNI.^13 14^ Moreover, we should consider whether a highly subjective and poorly reproducible assessment is appropriate to determine our allocation of life-saving treatments.^15^ Hence, criteria requiring repeat assessment act as a barrier to initiating additional therapies such as MRA or ARNI,^7^ which are regarded as ‘second-line’ due to the hierarchical framework which places greater emphasis on therapies based on the chronological sequence in which the trials were performed. There is no logical basis to assume that drug classes trialled earliest would be the most beneficial, yet this is what guidelines imply. Therefore, if we are to make progress, future guidelines must address these limitations and incorporate the Four Pillars of Heart Failure into a comprehensive disease modifying programme for all people living with HFrEF.

## Learning lessons from ARNI

The PARADIGM-HF trial showed that a combination of angiotensin receptor blocker and a neprilysin inhibitor (sacubitril-valsartan) was superior to an ACEi in preventing cardiovascular deaths or hospitalisation for heart failure (HR 080, 95% CI 0.73 to 0.87) and reducing all-cause mortality (HR 0.84, 95% CI 0.76 to 0.93).^16^ Despite overwhelming efficacy which led to the trial being stopped early, the utilisation of ARNI in the real-world has been suboptimal, with less than 1% penetration in eligible patients. The reluctance of physicians to prescribe ARNI may in part be that, unusually, the recommendations are based on a single trial, studied in an ‘A+B vs C’ fashion. Furthermore, the comparator was a submaximal dose of enalapril compounded by lower blood pressure in those allocated ARNI suggesting undertreatment in the control arm.^16^ It has also been suggested that the trial was additionally biased in favour of the novel agent due to a double drug run-in period of unequal times, in which those randomised to ARNI had already received an ACEi and were therefore pre-selected (20% of patients were lost during the run-in period).^17^ Drug doses are related to outcomes in HFrEF^18–20^ and the HR for the composite outcome in PARADIGM-HF between sacubitril-valsartan and enalapril was similar to the comparison of high and low dosing of lisinopril in the ATLAS trial.^21^ However, post-hoc analysis has shown that the point estimates for the benefit of low dose ARNI compared with low dose ACEi were identical to the point estimate of the overall trial,^22^ and real-world data have shown clear improvements in outcomes, symptoms and quality of life compared with standard of care ACEi.^23^

Another difficulty employing ARNI across the board includes the wash-out period required following cessation of ACEi due to risks of angioedema. Although not insurmountable, such requirements challenge heart failure programmes facing reduced face-to-face appointments due to service redesign and the current pandemic. Hence, if the benefits of the activity are perceived (whether correctly or incorrectly) to be minimal, physician inertia may prevail. To counter this, initiating ARNI at the point of diagnosis would mitigate the risk of inertia while also providing a more effective treatment to patients during the period of highest risk.

## Implementing novel agents into heart failure pathways

The efficacy of SGLT2i in addition to standard therapies for people with HFrEF has been confirmed with consistent and near identical 25% risk reduction of the primary end point of cardiovascular death or hospitalisation for heart failure from both dapagliflozin and empagliflozin.^10 24^ Both trials also demonstrated a slow of decline in renal function; EMPEROR-Reduced showed a 50% relative risk reduction for the composite renal endpoint^10^ (although this was non-significant in DAPA-HF).^24^

It is anticipated that more than four out of five people with HFrEF in contemporary registries^25^ will be eligible based on the inclusion criteria of these trials. The beneficial effects on renal outcomes are particularly attractive in a disease process typically associated with progressive decline of kidney function which often prevents the initiation or intensification of renin-angiotensin system inhibitors. Furthermore, SGLT2i are safe, with a low incidence of serious side effects (no patients without diabetes developed ketoacidosis in DAPA-HF or EMPEROR-Reduced), a lack of dosing considerations and minimal effects on blood pressure.

Given the somewhat simpler approach taken in trials of SGLT2i, the ease of use and clear benefits, it is likely that uptake among physicians will be enthusiastic, although it is yet unclear how these agents might fit into our current practice. There is a risk that SGLT2i become an additional agent for patients who do not ‘respond’ with conventional pharmacological therapy, rather than a fundamental Pillar of Heart Failure. The totality of the available evidence suggests the benefits of SGLT2i are consistent across subgroups, including diabetes status, baseline ARNI and symptoms. SGLT2i must therefore be regarded as a unique class of medication with a novel mechanism of action to be used in all eligible patients.

## The possibilities of a comprehensive approach

The four drug classes are complementary to each other and cross-trial comparisons have shown that a comprehensive disease modification strategy beyond the treatments which most patients receive (ACEi and beta-blocker) with ARNI, MRA and SGLT2i are associated with improved outcomes. A typical patient aged 65 years can expect to live an additional 5 years if receiving a comprehensive strategy with the Four Pillars, compared with conventional therapy.^26^

## Inertia: know your enemy

Drugs, trial design and side effects aside, the key obstacle to therapy intensification is physician inertia in patients who are deemed to have stabilised or ‘responded’ to treatment. For some with a recent decompensation this might be appropriate,^27 28^ but the relevant clinical trials were carried out in ambulatory patients receiving stable doses of previous generations of medical therapy, most of which had class II symptoms.^10 16 24^ Of particular relevance to SGLT2i, a striking result from DAPA-HF was the early benefit from dapagliflozin, with a reduction in worsening heart failure events observed within 28 days.^24^

## Simplify to progress

**Figure 1:**
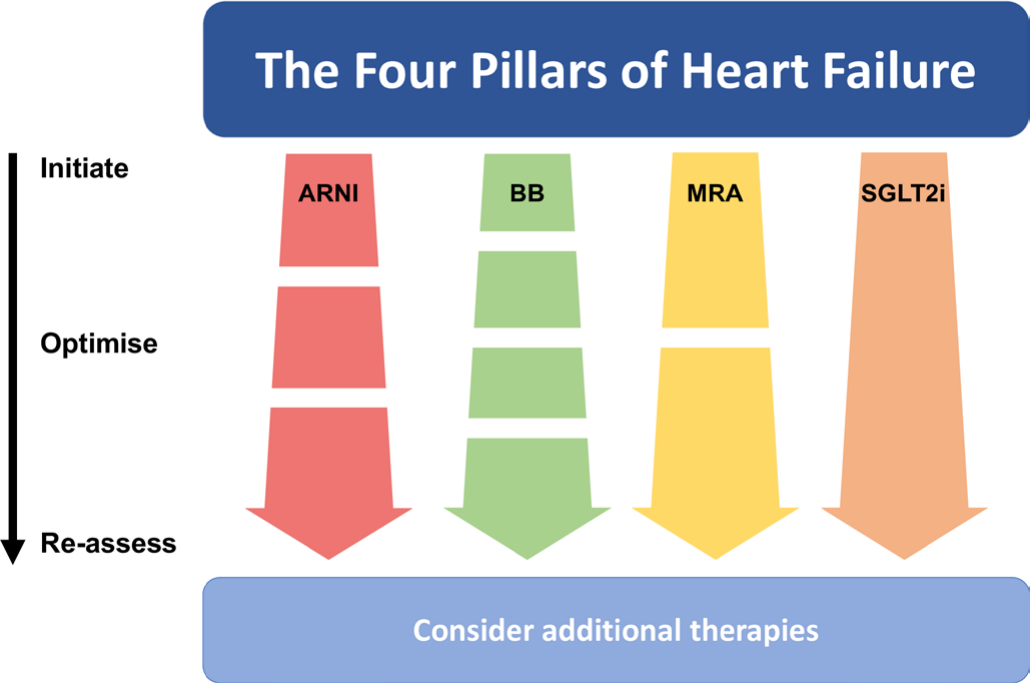
Initiation and optimisation of the Four Pillars of Heart Failure. All agents are initiated in parallel. This is followed by up-titration in one, two or three steps, as required. Additional therapies are then considered as a final step. ARNI, angiotensin receptor-neprilysin inhibitors; BB, beta-blocker; MRA, mineralocorticoid receptor antagonists;SGLT2i, sodium-glucose co-transporter 2inhibitors.

We propose a novel conceptual framework for the implementation of pharmacological therapies in HFrEF, in which the Four Pillars of Heart Failure are introduced in parallel, very early in the patient pathway with subsequent optimisation of dosing where required (figure 1).^24^ The rate of dose increments can be tailored to the patient and the service. For most patients, low dose ARNI and SGLT2i could be started simultaneously, followed within a few days by low dose beta-blocker and MRA, followed by up-titration. While others have suggested that initiation with beta-blocker alongside SGLT2i might be more optimal,^29^ we believe that the exact sequent of initiation is unimportant, as long as all Four Pillars are introduced within the first few weeks of diagnosis—although with the lack of dosing considerations and clear evidence from randomised trials of an early beneficial effect,^27^ SGLT2i are an obvious first choice.

This approach is at odds with current practice and a fundamental shift away from the linear approach advocated by guidelines, including the recent technology appraisal of dapagliflozin from the UK.^30^ Nevertheless, it remains the case, that very few patients become asymptomatic with even optimal doses of disease-modifying therapy suggesting that slow up-titration, followed by a decision to embark on the next step based on symptoms is folly.

It seems likely that impairment of renal function following up-titration of ARNI and MRA will become lesser concerns following the introduction of SGLT2i into care pathways, however, real-world data are vital to confirm the safety and feasibility of this approach. Those caring for people with heart failure must be cognisant that early increases in creatinine with SGLT2i are transient and be reassured that in the long-term the less rapid decline in renal function in patients receiving SGLT2i will allow more complete renin-angiotensin system blockade.

## Conclusion

The introduction of SGLT2i to the treatment of HFrEF is a chance for us to revisit whether current guidelines for the treatment of HFrEF are fit for purpose. Many patients have waited for weeks or months before ever seeing a cardiologist and receiving a diagnosis, and further delays to treatments have the potential to cause great harm. Moving forwards, we must recognise heart failure for what it is, an incurable disease with a mortality rate similar to many forms of cancer, where any delays cost lives. Once we have done so we need to implement pathways that offer rapid initiation and, where required, up-titration of life-extending therapies.
